# Analectic

**Published:** 1831

**Authors:** 


### Miscellaneous Intelligence.

### ANALECTIC, ANALYTICAL, AND ORIGINAL.

### I. Analectic.

1. *Case of Amnesia.*—By S. Henry Dickson, M. D., Prof. Institutes and Practice of Med. in the Med. College of South Carolina.

In May, 1829, I was requested to visit, in consultation, J. W——. He is a man of short stature, ruddy face, full habit, about fifty-five years of age, cheerful, talkative, quick, impatient; has been engaged in mercantile business; lives freely, but not intemperately. Some weeks previously, having eaten a very hearty dinner, he was seized in the afternoon, while occupied at backgammon, with a degree of mental confusion, occasioning him to rise from the table hastily, and walk about with his hand on his forehead in silence, and with an air of entire absence. This abstraction increasing, soon amounted to a total insensibility of external objects. A physician being sent for, he was bled largely, and gradually recovered, in a partial degree, his faculties. His situation seems to be in many respects unusual, and his case presents some curious pathological phenomena. He retains his muscular strength in great measure unimpaired; there is indeed no more emaciation or debility than must of necessity follow from restrictions in diet and remedial depletion. His appetite is good, and his digestion, for the most part, easy and natural. His sleep is composed, and not morbidly profound. His memory, and more especially his power of recollection, are affected. He is at a loss for words, but not always, for he will occasionally pronounce a whole sentence without hesitation. He seems to have lost the convential connexion between *an idea* and *the word* denoting it. I infer this from the following circumstances:—

He gives us to understand that he always remembers *the thing* aimed at, though he cannot express it. "I *know* a great many things—I cannot *speak* any thing"—is a phrase often repeated by him. He reads much, but says he does not understand, nor does he, in reading aloud, utter the correct words with certainty. He was very fond, for a time, of copying. He wrote a good hand, and seldom failed to write the proper word, but declares that this also he did without understanding. The difficulty seems to be, in both these instances, that he is unable, from extreme deficiency of retentiveness, to remember the first part of a sentence long enough to connect it into meaning with the conclusion. Some words he always failed to recollect. He was fond of molasses, and used it frequently with water to quench his thirst; but he could never remember the word, nor could he be brought to utter it, or take it from another person by suggestion, however frequently and distinctly repeated. He carried the word written out fairly on a card in his pocket, and would make, from time to time, the most strenuous efforts to order his servant to hand him the article, but in vain. He was much chagrined at this. "Why," said he to me, "can I not speak that word? When my friends visit me, I can order for them wine, ale, porter cider, brandy—but this I cannot speak."

He fancies, when he does not comprehend what is said to him, that he does not hear it, and complains much of this supposed defect. But that his hearing is perfect, is proved by a thousand instances of acuteness in this particular. He hears a distant sound, the ringing of a bell, the striking, and even the ticking of a clock, as well as any body about him.

He mistakes sometimes the names of his daughters, calls a day a week, drops a syllable, uses a word entirely inappropriate, or a set (though very rarely) of unconnected syllables, and becomes confused under a consciousness of his mistakes. This is apt to render him low-spirited; he is not uniformly so, however—he still loves a joke. While under a course of medicine, his wife, at dinner, happened to forget something which she wished to tell him;—rising hastily from the table, he ran off for a powder to give her, "*to make her remember.*" This he told me afterwards with great glee.

It is further remarkable that his perception and recollection of numbers have always been clear, at least comparatively and notably so. He reads and speaks of numbers accurately. An advertisement being placed before him, "———'s Lottery Office," he could not read the name, though, as he exclaimed impatiently, "he knew the man well!" But the list of prizes ranged beneath the caption he could run through fluently. He would remind his friends of the precise dates at which notes in his possession were to become due, and make all the necessary preparations concerning them; and this at a time when he could not write his own name unless from a copy, nor with uniformity utter it when shown him written, nor remember it regularly when he wished. A date he could at any time make out, but not always the month referred to in words.

In the above detailed case, it is not easy to refer the various phenomena presented, each to its own proper cause and origin. The *perception* of objects seems to be irregularly defective—not, however, from any lesion of the external senses, but from something peculiar in the condition of the brain. The patient, however, is unwilling to allow any such defect or perception as is here supposed. He contends that "he cannot remember—he cannot speak what he does perceive and understand." The only exception which he is ready to acknowledge, is found in the sense of hearing, which he believes to be dull, but which is in reality abundantly acute. Shall we account for all the apparent defect of memory by this imperfect perception? The less vivid the impression made on the material organ of thought, the less permanent it will be, of course. How then shall we explain the difference between the various sets of objects presented to him? *Words* were most frequently forgotten or mistaken,—*numbers* almost always promptly comprehended and recollected. Is it because the idea of a number—the impression made by it on the brain—is more intense, more clear, more deeply stamped, than that of a word; as a figure of definite form, and bounded by lines and angles of mathematical precision, is more distinctly and easily remembered than an uncertain shape or a shadowy outline?—*Amer. Journ. of the Med. Sciences.*

2. *M. Berard, senior, on the Veins.*—The June number of the *Archives Generales*, contains a memoir upon one point of the anatomy and physiology of the venous system; the mechanism of the accidental entrance of air into the veins; and the effects of the elasticity of the lungs.

M. Berard begins by remarking that an artery divided transversely, is said to remain open, while a vein treated in the same way, collapses, but thinks that he has ascertained that the veins have a greater tendency to remain open than closed; the reason of this permanency of the calibre is not to be found in the structure of the vein itself, but in its connexions with the surrounding parts. He speaks of the long known permanency of the channel of the longitudinal sinus of the dura mater, and mentions the gaping orifices of veins discoverable on the surfaces of cuts in the liver.

If the vena cava be opened above where the pericardial membrane is reflected upon it, it will be seen that the vein does not collapse; its parieties remain separated, although the blood contained in it escapes; it is kept dilated by the fibrous prolongation of the pericardium upon it. If the vein is freed by dissection from those adhesions, it collapses readily. The two subclavian veins, also, are kept in a state of perpetual dilatation, by means of aponeurotic fibres descending from the neck.

Mr. B. now proceeds to apply this anatomical knowledge to the explanation of a surprising and dreadful accident that occurred some time ago during the performance of a surgical operation on the neck, in which the patient suddenly died under the hands of the operator. The sudden admissiou of air into the veins, and its rarefaction in the auricle, put an immediate stop to the action of the heart. Seeing the frequency with which surgical operations on the neck are performed, and the extreme rarity of such an accident as the one above mentioned, one might be induced to suppose that there must have been some peculiar physical circumstance in the case. But if a vein be kept open by its connexion to the parts which it traverses, there is no occasion to refer to a suction power of the auricle or of the thorax alone, in attempting to explain the occurrence; for if the vein were capable of collapsing, the pressure of the atmosphere would compress its sides and prevent the admission of air into its cavity.

The state of tension or dilatation of the veins of the upper part of the body, is not confined to the cava superior, the subclavians, or their junction with the jugulars. If the axillary vein be examined from the scalenus muscle as far as the hollow of the armpit, it will be found to present a canal, whose parieties, as they are attached externally to an aponeurosis which descend from the clavicle and covers the subclavian muscle, can scarcely approach the axis of the vessel. M. Cruvichier was struck with this arrangement in examining a specimen which M. B. had prepared for his inspection. M. B. also exhibited a similar conformation to M. Hourmon, in the large veins situated near the transverse apophyses of the first cervical vertebræ, and in those that pass from the neighborhood of the shoulder to the inferior part of the internal jugular. It is also to be found in the layers of the temporal aponeurosis. M. B., after alluding to the similar state of things in the longitudinal sinus and the venous channels in the diploe, proceeds to inquire whether a like condition may not exist in the veins that are below the heart. He speaks of the vena cava as closely attached by means of a fibrous structure to the circumference of the opening by which it passes through the diaphragm, and remarks, that just below the diaphragm the cava receives the hepatic veins, which remain dilated after excision. He does not know whether the inferior cava remains dilated in the place where it passes through a portion of the liver; we think we may venture to say that we have often seen the sides of the vein nearly collapsed. M. B. could find the arrangement under consideration no where else except in the spine and pelvis. The veins of the pelvis presented an appearance scarcely differing from that of the sinuses of the dura mater. The large trunks of the hypogastric veins adhere to the fibres through which they pass, and are thus kept dilated by the upper pelvic aponeurosis.

This arrangement of the veins, according to M. Berard, facilitates the entrance of the blood into the lungs during inspiration. He is confirmed in this opinion by reading the memoir of Barry, by the report of Dumeril and Cuvier, on the alternate contraction and dilatation observed in the tumour of certain cases of *spina bifida*, and by the arrest of some venous hemorrhages by respiring freely and regularly, a fact made known twenty-four years since.—*N. A. Med. and Surj. Journal.*

3. *Catalepsy.*—A case of this strange disease having recently appeared in the Royal Infirmary of Edinburgh, the clinical remarks of Dr. Duucan have been collected and published in a weekly contemporary. We shall take a short notice of this case, the more especially as one not very dissimilar is at the present moment under our care.

The northern patient was a young woman 25 years of age, who became affected with catalepsy, about the first of February of the present year, and was received into the infirmary on the 5th of the same month. She was first discovered sitting in the kitchen like a statue, stiff and insensible—yet her limbs yielding easily to any force which was applied, and remaining in a position they were put. When received into hospital, there was a complete loss of voluntary motion occurring in paroxysms, and continuing about ten minutes at a time, during which the limbs retained a certain degree of flexibility, but resisted to a certain extent, the application of external force. During the fits there were slight writhings of the muscles—the eyes were not turned up, as in epilepsy, but rolled about, the pupils dilating and contracting. Before the commencement of the paroxysms she feels a fluttering about the heart, general faintness, and heaviness about the head. After the paroxysm is over, she feels a universal soreness of the limbs, as epileptics do, attended with a severe pain in a certain part of the spine, which is tender to the touch. The tongue was white, pulse 70 in the intervals; but rapid and feeble during the fits. *The catamenia had appeared at the proper period, before the commencement of the disease, but were suddenly suppressed by cold.* Some inhuman sceptic had torn two pieces of skin off her hands in one of the paroxysms!

The case continued much the same, not at all relieved, till the 15th of February, when severe pain was complained of in the right iliac region, which, though reasonably considered as nervous by Dr. Duncan, was treated by leeches, lest inflammation should actually exist.

During the cataleptic paroxysms, she was, of course, free from pain; but the moment they were over, she screamed out from the violence of the agony. The iliac region was extremely tender to the touch; but no tumor was perceptible. The leeches bled freely—a fetid enema was thrown up, but speedily returned. Powerful anodynes were given both by the mouth and anus, yet without any effect. A warm bath and a still more overwhelming opiate allayed the agony. In the course of little more than twenty-four hours, she had taken 125 drops of Battley's sedative liquor, 120 drops of common laudanum, two grains of solid opium, besides castor, ether, valerian, and most of the stinking drugs in the pharmacopœia. These and other circumstances induced Dr. Duncan to conclude that the disease was of a purely nervous character, and, in fact, that it was one of those singular and strange forms which hysteria occasionally assumes. After this the complaint took on another new but temporary form. On the 3d of March, the patient received some unpleasant intelligence, and requested her dismissal. This being refused, a very severe paroxysm occurred, and lasted three-quarters of an hour. Several of these attacks came on in the course of a few hours.

Dr. Duncan appears to have taken considerable pains to prove that, contrary to the doctrine of Cullen, Catalepsy may be real, and not feigned. We have not the smallest doubt of the reality of the disease in the foregoing, and in many other cases. We are, at this moment, attending a young lady who has cateleptic attacks every day, and many times during each day. They are generally so transient as to be mostly imperceptible in company—and her object is to conceal them. They escape notice, except upon particular occasions. Thus, if she is reading, or playing on the piano-forte, the sudden cessation of voice or action is remarked, of course. But if she is merely sitting in company, or joining in general conversation, it is ten to one if the cataleptic suspension of volition be perceived. Once in six or seven days she is seized with a convulsion, in all respects answering to pure epilepsy. The attack commences with a shriek—she falls down—struggles violently—becomes hideously distorted—bites her tongue—and requires two or three attendants to constrain her contortions. She then falls into a sound sleep, and awakes sore and rather poorly, but unconscious, except by these sensations, of what has passed. The complaint has been gradually increasing in violence and frequency, from the age of six to eighteen years. The catamenia have only appeared once, and have not since recurred. Moving in a high sphere of life, this young lady has had the very best—and perhaps the very worst advice which England could afford. Not the slightest impression has ever been made on the distressing malady—on the contrary, it has progressively augmented in force. Although the intellectual facultics have suffered less than might have been expected, they have not escaped uninjured. The memory is impaired, and application to some particular studies is greatly abridged. She dares not indulge in music—and she is incapable of making the slightest progrcss in arithmetic. She cannot perform even the most common operations in figures. She delights in history; but the impressions are like those made in water, or, at most, in sand. They are soon obliterated. The eyes are expressive—the pupils very large; the complexion exquisitely fair; the features beautiful; the temper mild; and the anxiety to be relieved from her malady intense. The intestinal secretions and excretions are exceedingly depraved, and the catamenia are stopped. * * * Her great object is to veil her attacks, and the cataleptic paroxysms usually escape the observation, except of her parents or intimate relations. If she is reading, for example, she will suddenly stop, for an instant—perhaps for half a minute or a minute; being, for that period, like a marble statue—and then she utters a kind of sigh, and takes up the word, or part of the word, where she had stopped. Considering the length of time which the complaint has obtained, we cannot, of course, form any sanguine hopes of recovery. The first object which we have in view, is to correct the alvine derangements and to reproduce the uterine functions. The result of the case we shall freely and candidly communicate to our readers.

In reviewing his case, on the patient's discharge from the hospital, Dr. Duncan observed that the symptoms and character of the malady had considerably varied during the progress of the complaint. These variations he was inclined to refer rather to a "modified form of hysteria than to any other disease." As the case proceeded, the paroxysms became more and more accompanied by gesticulations, singing, &c., instead of the rigidity of catalepsy. The form of hysteria approached the epileptic, as, in the paroxysms, the patient was totally devoid of consciousness or feeling. The remedies which gave most relief in this case, were those which are most useful in hysteria—antispasmodics, narcotics, and purgatives. The shower bath had been employed two days after her admission, and apparently with some benefit at first; but subsequently with mischief. Powerful purgatives brought away pitchy stools, and temporary relief followed. Indeed the purgation appeared more beneficial than any of the other remedial measures, till the new symptom of excrutiating pain in the abdomen set in, when leeches were applied. This pain was so torturing, that the accession of the cataleptic paroxysm was desirable, as a temporary insensibility to sufferings. The cause of these sufferings, Dr. D. was unable to explain—who, indeed, can hope to explain the mysteries of the nervous system in hysteria? One thing is certain, that depletion did not relieve this excruciating pain. Dr. D. alluded to those unaccountable tumors which sometimes show themselves in hysterical females about the groins, causing dread of hernia in the minds of medical attendants. He was convinced at any rate that these tumors were *internal*, or situated beneath the muscles constituting the abdominal parietes. In this case flatulence was very distressing. In one instance, after a fright, the spasms became actually tetanic, and lasted four hours, during which time, though the muscular system was in a state of cataleptic rigidity, she sang hymns and repeated the Lord's prayer in a perfectly rational manner. During the eruptive fever of the smallpox, the paroxysms went on; *but were suspended during the presence of the eruption itself*. This was a fortunate circumstance, as a continuance of struggles, during the eruption of pustules on the surface, would have been most distressing. The alcoholic extract of nux vomica was afterwards given in large doses, so as to induce a considerable degree of narcotism. From this time she gradually improved till she was discharged—but whether this improvement resulted from medication, or one of the freaks of the malady, it would be difficult to say. One thing is certain, that, in less than three weeks, she returned in statu quo, and remains in the infirmary. We shall report on both the cases mentioned in this paper on a future occasion."—*Med. Chirur. Rev.*

4. *Efficacy of Cold Affusions in Nervous Affections.*—A case very illustrative of the power exercised over nervous disorders, by the shock produced on the surface by cold affusion, is reported as having recently occurred in one of the Parisian hospitals. The patient, a juggler, was found in his room in a state of insensibility. Shortly after he was carried to the hospital he became delirious. His face flushed, his hair erect, his eyes wild, and speech incoherent. Yet were there no symptoms of febrile excitement distinguishable in the pulse, or the condition of the skin. Speculation was fruitful as to the cause and nature of this condition of body, and some suspicions were entertained, that the gentleman was only engaged in a professional exhibition. The medical officer of the institution, however, soon recognized it as a case of purely nervous character, and directed cold affusions, from head to foot, of water at the temperature of 18 deg. Reaumur. The delirium yielded to this remedy immediately, and the patient, next day, was walking about the ward.

It is no novelty to find nervous complaints disappearing before this remedy; but the influence it is capable of exerting in hysterical disorders, which are so common and so troublesome in young and old females, is not, perhaps, so familliar to every practitioner. No class of disease is so troublesome to the medical attendant, as none is more difficult to be borne by the patient, than that denominated *nervous*. These diseases ape so many others of different character, that an inattentive observer is apt to commit himself by an erroneous opinion, and be led through a series of prescriptions of various and discrepant remedies, all with the same result—disappointment and vexation. At the same hospital as that above referred to—the Hotel Dieu—a case is reported in which cough, slight hæmoptysis, palpitations, obstinate rejection of food, &c., led to the administration of a succession of antispasmodics and sedatives, all to no other purpose than to excite surprise that they should not arrest the symptoms. Suspecting at last that this might be an instance of those anomalous hysterical affections which are so often mistaken by ordinary observers, the physician ordered that affusions of water at 18 deg. Reaumur, should be directed on the head, whilst the body was immersed in a bath of a temperature 8 degrees higher:—a bad practice certainly for hæmoptysis and cough depending on common causes, but a good one, as the result here proved, where these symptoms are purely nervous.

The mode of using the cold affusions in such instances, varies with the circumstances of each case, and the taste, habits, and information of each medical attendant. By some the shower bath is preferred; others pour water from a pitcher directly on the head; and still others interpose on the crown, a very large sponge, an expedient the advantage of which is obvious. Dr. Johnson recommends the shower bath, with the precaution, in commencing its use, of letting the patient stand with the feet in a tub of warm water. This precaution is particularly advisable with young females of delicate constitution, but with all it will add security to an effectual means of cure, and detract somewhat from the aversion with which this remedy is too often anticipated.—*Bost. Med. and Surg. Jour.*

5. *Homicidal Monomania.*—We derive the history of this case from the *Annales d'Hygiene Publique et de Medecine Legale* for July last, in which it was published as a communication to Dr. Marc, from Professor Grossi, of Munich. B., a farmer in the neighborhood of Landshut, in the kingdom of Bavaria, more than 70 years of age, but whose powers, both physical and mental, were still quite active, had been married a second time, and became the father of two children, one of whom was eight, and the other ten years of age. His neighbors, and the persons in authority in his district, had always known him to be an upright, laborious, and intelligent man. He had even been often chosen as arbitrator in disputes in matters of rural economy.

Prior to the last war, of which his country was the theatre, and the augmentation of taxes, B. passed for a man in easy circumstances; but these evils, as well as the portions he was obliged to give up to his children by his first marriage, lessened greatly his fortune. B. became sad, and was continually complaining to his friends of the hardness of the times, and of the impossibility of farmers paying taxes and meeting house expenses. His confidence in the clergy and his religious zeal were abated. He often, however, had religious scruples, and sought by frequent vows to restore calm to his bosom. Having no longer any reliance on the priests in his neighborhood, he addressed himself to the curate of a remote district, who thought he perceived a certain disorder of his intellectual faculties, a state which his neighbors declared they had never observed. The people who lived under the same roof with B. affirmed also that they had never any occasion to complain of him; only it was remarked that he treated his wife with somewhat less mildness. As to his children of the last marriage, he continued to display to them the same affection as before. His sleep had become less tranquil: but this circumstance was not attended to, it being attributed entirely to advanced age. At last the uneasiness of mind which his reflections on the derangement of his domestic affairs gave rise to, became so aggravated that he spoke in a tone of the deepest despair, and seemed to lose all hope of the temporal and eternal welfare of his children who were under age. Their spiritual well-being was the special subject of his solicitude.

For a long time a misunderstanding existed between him and his nearest neighbor, who had in fact done him wrong; but this was the only person with whom he could not agree. This enmity on the part of B. acquired a singular force, in consequence of this state of feeling, and even assumed a peculiar character. B. regarded his neighbor as the principal author of all his evils, accused him of wishing to seduce or corrupt his children and servants, had frequent altercations with him, and even uttered, several times, threats against his life.

For a year past, B. had taken into his service a man who had enjoyed a good reputation, and whose duties consisted in superintending the labors of the farm. Soon, however, B. complained of his carelessness, his pride, and of his unnecessary expenditures, such as, for example, his purchasing harness with brass mountings. He accused him also of having been too late in putting certain fields under cultivation, and of paying no attention to his injunction not to visit, during his leisure hours, the neighbor who was an enemy to his master

B. accordingly determined to send away his servant at the expiration of the customary period. He gave him due notice, and paid him without evincing the slightest deviation from propriety. On the evening before the day of the man's departure, he settled his account, addressed no reproach to him, and the day had passed tranquilly, without any suspicion being excited that B. entertained the slightest intention of committing the act which he did the next day. B. went to bed earlier than common, and did not undress himself, an omission which was sometimes observed when he did not feel easy in his mind.

Though it was winter, his servants rose at four o'clock in the morning for the purpose of thrashing in the barn; and the man who was discharged, but according to custom could only quit the house after breakfast, aided in this operation. On this day B. was also up earlier than usual, and smoked his pipe immediately after rising. It was while thus employed, as we are constrained from the sincerity of his declarations to believe, that he conceived the idea of revenging himself on his servant, who, in concert with his neighbor, had done him wrong, and had, among other things, carried from the barn of this latter weevils into his. He thought that he ought to avail himself of the short space of time, during which the man was yet to stay, to wreak his revenge on him. In fact he declared that the idea of the injuries which this person had done him, occupied his mind to the exclusion of every other thought.

Having got possession of a gun, which, according to the testimony on the trial, was already loaded, B. went to the porch, and availed of the imperfect light to conceal himself against a clothes-press, before which the servant must necessarily pass in order to go from the barn to the public hall. But this latter returned in company with all the servants, so that B. could not put his design into execution without danger of wounding some other person. He consequently kept quiet, and went up stairs to his room. There was in the floor of this latter a *judas*, by which he could see his people during their meals. The obnoxious servant was in the middle, and near the opening. More than ever intent on his dreadful scheme, B. raised the lid which covered the *judas*, and armed with his gun, kneeled down, as he declared, in such a position as to be sure of his aim, hurt no other person, and not be seen. In fact his daughter, who was at the table, and whose looks were directed to the ceiling, could only see the end of the murderous fire-arm, which she mistook for an iron pipe. B. took aim with great care at his servant, and mortally wounded him in the chest. Before committing the murder, he adopted the precaution to shut the door which led outwardly from the room in which he was, and to take out the key, so as not to be prevented either from executing the act of which we have just spoken, or that which he afterwards committed on his two younger children, who slept in an adjoining room.

Without paying any attention to the outcries of the persons present, nor answering the questions put to him, he went directly to his children's room, and seizing a hammer which had been there for a long time, killed his son while this latter was asleep. He went immediately afterwards to the bed of his daughter, whose entreaties and feeble resistance were alike unavailing to prevent him from striking her several blows on the head, until he thought her dead.

When the ministers of justice reproached him for the atrocity of this conduct, and especially for not allowing himself to be softened by the prayers of so interesting a child as his daughter, his reply was that, doomed on account of the murder of the servant man to perish on the scaffold, he thought it best to preserve his innocent children from the world's seductions, and especially from those of his neighbor, who had already made a gambler of his oldest son, and encouraged him in disobedience, rather than to expose them to suffer both in their spiritual and temporal welfare. He frequently repeated this declaration, and always with the same coolness, and he often deplored, when in prison, the state of his daughter, after having learned that she had not sunk under her wounds.

B., in reference to his homicidal deeds and attempts, made the following declarations, especially remarkable in a pathological point of view: they were in reference to two assassinations committed in an adjoining district about a year before the events now related had transpired.

An apprentice shoemaker wishing to purloin a watch from the shop of a watchmaker, succeeded under divers pretexts, in sending off the owner and seizing a watch of small value. At the moment of escape, the watchmaker returns, a struggle ensues, the robber, armed with a hammer, kills the shopkeeper, and a person passing by, attracted by the cries of this latter, on attempting to seize the wretch undergoes the same fate.

B. compares his situation to that of the apprentice shoemaker. I was, said he, in the way of killing, and, like him, I could not stop myself, though my motives cannot be compared with the meanness of those which induced the apprentice to commit a double homicide.

The inhabitants of the house and neighborhood presented themselves at different times before the door of the room in which B. had shut himself up. He had several short conversations with them; but they could not induce him to open the door. He dressed himself very warmly, as the nature of the season required, took some money and his pipe, with all the et ceteras for smoking, and without opening the door through which a person could pass into the children's room, he went into a corridor leading to the yard. When there he directed a servant to harness two horses to a sledge, in order that he might go to town; he bade his people farewell, without, however, showing the least compunction, and gave the signal for departure. He waited even for the moment of setting out to throw to the persons who were near him the key of the door, but it was only after the lapse of half an hour that he informed the driver of the murder of his children. Having reached the seat of justice, he had himself taken to the house of a physician acting as medical jurist, left the sledge with the greatest coolness, bade farewell in a few words to his servant, had himself conducted to the judge's office, and there made, without exhibiting the slightest compunction, a declaration of the acts which he had just committed, and begged that the trial might be hastened, as well as the carrying into effect of his sentence of death. He several times testified the same wish during the trial.

When B. learned in prison that his daughter had not been destroyed, and was even convalescent, he spoke several times of her with marked interest; but always displayed a wish for her death, in order that she might no longer be exposed to the temptations of the world.

I, says professor Grossi, consider B. as having been in a state of insanity. My opinion was founded on the following circumstances: his having more and more despaired of the temporal and spiritual welfare of himself and his children; the relation which existed between him and his neighbor having become a fixed and dominant idea, carrying with it the thirst for vengeance; his having performed many religious acts foreign to his habits; the sudden execution of his projects of revenge on his servant; and finally, the murder itself of his own children, which was perpetrated immediately after that of the servant. These various acts seem to me to indicate a partial delirium, with a subversion of the affective feelings. On these grounds B. was not considered by the judiciary as an assassin. He was secluded in the house of correction at Munich, in which, in despite of the most assiduous care, he sunk at the end of the year into a state of evident dementia or fatuity.—*North American Med. and Surg. Jour.*

6. *Case of Inflammation of Vena Cava Ascendens.*—Dr. J. Hourmann relates in *La Lancette Francaise*, the following example of this affection, which is interesting as exhibiting the immense resources of nature, and as confirming many points in the history of phlebitis. The subject of the case was a porter, of strong constitution, and in the habitual enjoyment of good health. Some weeks before his entrance into hospital, he fell, and struck, violently, the right side of his chest against a step. Leeches were applied to the injured side, but a dull pain remained. About fifteen days afterwards, he perceived a swelling of the right foot, and soon after of the whole lower limb of this side. He experienced also an uneasiness in the groin of the same side, and then the left limb, which had previously been unaffected, began to be infiltrated. In this condition the patient entered the hospital He had no marked functional derangement of the viscera, and he did not complain except of the swelling of the limb. M. Louis immediately pronounced the disease to be phlebitis, which had commenced in the right crural vein, and which had successively extended to the external iliac, the primitive iliac, and lastly, to the vena cava ascendens. Before the vena cava became affected, there was no obstruction to the circulation of the left limb; but as soon as the inflammation extended to the former, the latter became infiltrated. At this period none of the veins of the limbs were very remarkably prominent; however, on examining with the fingers, in the course of the saphenas, some renitence was perceived; the veins of the anterior of the abdomen did not exhibit any unusual appearance. The diagnostic of M. Louis was soon confirmed. In fact, in a few days many veins were observed to become developed, arising from the groin, and ramifying over the abdomen towards the chest. One of these on each side passed to the neighborhood of the armpits. The size of these veins was at first small, but they soon became enlarged, and the two which ran to the armpits became as large as a goose-quill. When this vein was compressed with the finger, the blood was observed to stagnate below the place of compression, and to accumulate in the groin; when the vein was emptied by pushing the blood towards the chest, the blood rushed from the groin towards the point of compression, and instantly filled the vessel. Once only the blood was observed to flow in an opposite direction. Did this depend, asks the relator of the case, upon an obstruction to the passage of the blood through the cavities of the heart, and a reflux into the vena cava? From the appearance, it cannot be doubted that there existed an obstacle to the passage of the venous blood from the inferior extremities by the ordinary routes, and of the establishment of supplementary circulation. The blood arrived at the groin not being able to obtain a passage through the crural vein, escaped by the orifice of the saphena, and from it, reversing by its force the natural course of that which came from the abdominal parieties, it passed towards the heart through the superficial veins; one of them uniting with a thoracic vein, discharged the fluid into the axillary trunk, and the vena cava descendens poured it into the right auricle. The patient experienced few inconveniences; there were some premonitions of a pleuro-pneumonia, which excited fears for a moment of the common consequences of phlebitis, the passage of pus into the circulation. But being promptly met, these serious symptoms were soon dissipated. M. Louis restricts himself, as regards active treatment, to bleeding, often repeated. By these means, and the admirable efforts of nature, the œdema almost entirely disappeared, and the patient was discharged almost perfectly well.—*Lond. Med. and Surg. Journ.*

*Chlorine an Antidote to Hydrocianic Acid.*—MM. Persoz and Nonat, have verified the favourable results which M. Simeon had obtained relative to the remedy which chlorine affords against prussic acid. They operated upon three dogs, upon the eyes of which a drop of prussic acid had been placed. Dividing the symptoms in three periods, namely, 1, uneasiness, 2. tetanus, 3, interrupted respiration, they found that when chlorine was applied in the first period, the relief was immediate, the respiration became regular, vomitings and alvine discharges occurred, the animal gradually regained its strength, rose unsteadily, and, in about half an hour, was as lively as at first. Applied at the second period, the symptoms were arrested, but the restlesness continued awhile; and though respiration was less painful, the convulsive movements continued for ten minutes, then occurred vomitings, &c., as before, and at the end of an hour, the animal was perfectly well. The two dogs thus treated, being tried next day with the same quantity of prussic acid, but without chlorine, died in a few minutes.

In the third case, all the effects of the prussic acid were produced before the chlorine was applied: the respiration had ceased for twenty-five seconds, and the animal was rapidly perishing; but the chlorine not only recalled it to life, but ultimately restored it to full vigor: the full effect only occurred, however, after some hours. Ten days after it was quite well, and the paralysis of the abdominal parts, which occurred in all, had in this case entirely disappeared.

After this, MM. Persoz and Nonant sought to ascertain whether the prussic acid, being absorbed into the vessels and tissues, the chlorine would follow and decompose it. Two dogs of equal strength were taken, the crural veins laid bare, and separated from the neighboring parts, and especially the accompanying nervous fibres; then a drop of prussic acid was put upon each vessel. The effects were instantaneous; a few drops of chlorine, (solution,) were let fall on one of the crural veins; the other animal was left alone. The first was as immediately recovered as it was injured; the second died directly. The first felt no inconvenience after some hours, except from the wound. Endeavors were then made to kill him, by putting prussic acid upon the eye, and upon the crural vein of the opposite side; but the animal only felt temporary inconvenience, and a few convulsive movements, and was very quickly at ease. Hence it appears that the chlorine administered beforehand is taken into the circulation, and is then an effectual remedy against prussic acid.—*Journal of the Royal Institution of Great Britain.—Lond. Med. and Surg. Jour.*

7. *Excision of a Carious Rib.*—A shoemaker, aged thirty-eight, was admitted into La Charitie, on the 23d of March, with a fistulous opening on the right side of the chest, leading down to the fifth rib. The latter, when examined by the probe, felt rough, denuded, and carious. A considerable quantity of puriform matter was discharged from the fistulous opening; the patient was debilitated, thin, and suffered from a troublesome cough, with expectoration of thick mucous sputa; but no positive sign of phthisis pulmonalis was present.

On the 24th of April, M. Roux proceeded to remove the rib. All the soft parts covering it were included between two semi-elliptical incisions, extending from the border of the axilla to near the sternum, and passing immediately under the mamma. By these incisions, and the removal of the integuments included between them, the rib was exposed for the extent of five inches; the limits of the carious portion ascertained, and the latter, four inches in length, cut out by means of the chain saw; another small portion near the sternum presenting a suspicious appearance, was also taken away. The pleura costalis adhered as usual to the inferior border of the bone, but above it was thrust inwards by a moderate collection of pus, which had no communication with the pleural cavity. The rib was quite carious, the superior border in particular and the internal surface being rough, deprived of its superficial laminæ, and chiefly affected about midway between its two extremitities. The wound was simply dressed, and for two or three days the patient appeared to be doing well. Then, however, dyspnœa and symptoms of pleuritis on the right side appeared, and death speedily supervened.

*Sectio Cadaveris.*—The right side of the chest contained a considerable quantity of sero-purulent fluid, with some flakes of recent lymph. The fluid was confined to the two lower thirds of the pleural cavity, the upper being closed from old and firm adhesions. The upper part of the lung contained many large and half-softened tubercles, principally situate opposite the second, third, and fourth ribs, which were all carious, and broke with the greatest facility. The pleura at this part still continued sound. The apex of both lungs was loaded with tubercles, chiefly of the granular kind; some of them were softened, others were not; there was nothing like a vomica. No disease was discoverable in other organs.

The reporter remarks that he is not aware of the operation having been performed more than once in France, which was by Richerand, in 1818. The patient died. Joshua Aymar, a surgeon of Grenoble, twice excised several carious ribs with success. The first patient was a woman, forty years of age, in whom the eighth and ninth ribs were diseased; the second a captain, whose fifth, sixth, and seventh were affected. M. Cittadini, has recently published five cases of successful excision of one or more ribs. In all he opened the cavity of the pleura, and one patien nearly died from the admission of air into it. In the present case of M. Roux's, the tubercles of the lungs must undoubtedly be considered as contributing essentially to the unfavorable character of the case and the fatal pleuritis. At the same time the result is calculated to point out the uncertainty that must always hang over this operation, and deter practitioners from engaging in it wantonly, or without the most cautious deliberation.—*Med. Chir. Rev., from the Journal Hebdomadaire.*

8. *Method of Cleaning Bones.*—Mr. J. W. West, of Northampton, recommends a combination of chloride of lime and subcarbonate of potash, in the proportion of one pound of the former to one ounce of the latter, added to two gallons of water, for the purpose of whitening bones and destroying the unpleasant odor generally issuing fr m them. He says that it rendered a cranium perfectly white, after having been in the solution not more than twenty-four hours.—*Lond. Med. Gaz.*

9. *Period of Puberty in Women.*–Mr. Roberton has given in the *North of England Medical and Surgical Journal,* the following table, exhibiting the ages at which three hundred and twenty-six women began to have catamenia.

Table.

| | | | | |
|---|---|---|---|---|
| In their 11th year | 6 | | In their 16th year | 54 |
| 12th | 12 | | 17th | 50 |
| 13th | 31 | | 18th | 19 |
| 14th | 60 | | 19th | 18 |
| 15th | 72 | | 20th | 4 |

This table was formed by questioning the pregnant women who came to the Lying-in Hospital, to deliver in their letters of recommendations as home patients. These women were generally in health, as appears by their walking, in an advanced stage of gestation, a considerable distance to the hospital. The circumstance of pregnancy is a guarantee, as regards the whole of the cases examined, as exemption from serious disease of the generative system. Every answer was rejected which evinced either a doubtful recollection of the fact, or which was reluctantly given in relation to the fact. The question was also put, not as if a matter of curiosity, but as connected with other questions necessary to be answered for admission as a patient. —*Lond. Med. and Surg. Journ.*

10. *Menstruation continued to the 94th year.*—A case is recorded in the *Ann. Univ. di Med.*, of a female aged 94, whose relatives were remarkable for their longevity, and who continued to menstruate from the 53d to the 94th year, and at present she is in perfect health. —*Ib.*

11. *Pregnancy occurring during Dysmenorrhœa.*—Dr. Ryan says, that two cases of dysmenorrhœa have lately fallen under his care, both aggravated by marriage, and both followed by pregnancy. One of these patients was twenty-three years of age, the other eighteen. Neither of them passed the membranous shreds, described by obstetric writers.—*Lond. Med. and Surg. Journ.*

12. *Salicine not an Alkali. Mode of Preparation, &c.*—We have already given some notice of this new principle, which has been found in the bark of the willow. We are now enabled to present other facts with regard to it, from a report made May 10th, 1830, to the Royal Academy of Sciences at Paris, by MM. Gay Lussac and Magendie, who were appointed to examine the memoire of M. Leroux, addressed to the academy in the preceding month of June.

It is known that in this memoir, M. Leroux claimed the discovery of a proximate principle in willow bark, which he called salicine, and which he conceived to possess alkaline properties. At the same time, he presented to the academy a substance which he considered to be the sulphate of salicine. It now appears that this principle has no claim to rank among the vegetable alkalies; that instead of saturating the acids, it is in fact decomposed by them, and loses the property of crystallizing, and that it contains no azote. It is therefore evident that the product named by M. Leroux the sulphate of salicine, can no longer be considered a salt. It is due to this gentleman to state, that the merit of discovering these facts is shared by himself with the committee.

The salicine of M. Leroux, is, when pure, in white slender shining crystals, very soluble in water and alcohol, but not in ether. Its taste is very bitter, and resembles that of the willow bark. The species of willow from which it is derived, is the *salix helix*, though it is not improbable that the bark of many other species, and among them some of American growth, will be found to contain it. The following is the process for obtaining it.

Boil three pounds of the willow bark, dried and reduced to powder, in fifteen pounds of water, holding in solution four ounces of carbonate of potassa. Strain the decoction, and when it is cool add two pounds of the solution of sub-acetate of lead. Filter the liquid, treat it with sulphuric acid, and complete the precipitation of the lead by a current of hpdrosulphuric acid. Then saturate the excess of acid by carbonate of lime, again filter, concentrate the liquid, and treat it with diluted sulphuric acid to perfect saturation. Finally, deprive it of color by means of animal charcoal, filter it while boiling hot, crystallize by two successive salicine operations, and dry the crystals in the dark. By this process about an ounce of salicine will be obtained. Conducted on a large scale, it would probably yield double the quantity. It might be considerably simplified.

In former numbers of this journal evidence has been given of the efficacy of salicine in curing intermittent fevers. Additional testimony is advanced in the report of the committee of the French academy. M. Magendie has seen fevers cut short in one day by three doses of six grains each. Besides the experiments of Dr. Miquel, before mentioned, others have been instituted at the Hotel-Dieu, by MM. Hudson and Bally, and by various other physicians; and all agree that from twenty-four to thirty grains of salicine are sufficient to prevent the recurrence of the paroxysm, whatever may be the type of the fever. It will be observed that this does not much exceed the dose of the sulphate of quinia.

The committee conclude their report in the following terms. "The discovery of M. Leroux is beyond contradiction one of the most important which have been made for many years in therapeutics. We are the more indebted to M. Leroux for the result of his labors, as many chemists, among whom are Rigatelli, Buckner, and Fontana, had previously occupied themselves with the bark of the willow, and believed that they had obtained its active principle in a state of purity. But it is evident, from the very terms of these chemists, that they have not succeeded in isolating the pure and crystallizable salicine, such as that which has been made known by M. Leroux."—*Archives Generales.*

13. *Wound of the Trachea and Œsophagus.*—We learn from the Medico Chirurgical Review for July, that Dr. Luder had charge of a man, aged thirty seven years, who cut his throat with a pruning knife. He plunged the crooked point of the knife into the neck at one side of the trachea, then pushed it under that tube and the œsophagus, and by drawing the instrument outwards, severed the alimentary and aerial conduits completely: the wound penetrating to the cervical vertebræ, without wounding any of the great vessels or nerves; the sterno-cleido muscles even escaped. The patient was pallid, deprived of speech, and could breathe only when lying on the abdomen, so that the blood could escape by the external wound. He rejected all assistance during the first day, and all future attempts by Dr. Luders, to aproximate the edges of the wound completely failed.

*Second day.* Much bloody mucus expectorated; was able to lie on his back, and made signs of hunger; some gruel and laudanum were thrown up per anum.

*Third day.* Free from fever; comfortable, excepting being hungry; some milk and egg were injected through a tube in the œsophagus into the stomach.

*Fourth day.* Better; by bending his neck forward, succeeded in swallowing a little milk.

From this time the patient convalesced; every day he could swallow better; the wound in the œsophagus healed, that in the trachea remained fistulous, and the patient breathes through a tube, which has now been continued for near two years.—*North American Med. and Surg. Jour.*

14. *Strangulation of the Spermatic Cord in the Abdominal Canal.*—In the Journal des Progrés for January, there is related a case from the Journal Hebdomadaire, of a man forty-three years old, who attempted to destroy himself by cutting his throat and removing his right testicle. He was placed under restraint at the hospital, and was doing well until the ninth day, when symptoms of strangulated hernia supervened; vomiting, tympanitic abdomen, clammy sweats, &c. &c., with painful swelling in the groin, and suppression of intestinal discharges. On pressure of the swelling, great pain was excited, and pus flowed from the abdominal canal. On the 10th day, the whole was laid open by means of a directory and bistoury. Pus immediately flowed out, and the tumefied spermatic cord appeared to dilate by a true expansion. The vomiting and nausea ceased, an abundant stool followed, and the wound healed without difficulty.—*Ib.*

15. *Extirpation of Cancer of the Rectum.*—We have already, in former numbers, given some cases of Lisfranc's operation for extirpating superficial cancer. A general view of the subject has been published by one of his pupils, M. Pinault, in 1829. We extract the following summary from the Journal des Progres for January.

That any attempt should be made to excise the inferior portion of the rectum, it is requisite that the index finger can be passed beyond the limits of the disease, that the extent of the induration may be determined; great difficulties would occur if the cancer had extended to all the tunics of the intestine, and especially to the surrounding tissues. A judgment may be formed of the severity of the disease by the mobility of the parietes of the intestine.

In operating, after the intestine has been emptied, and the patient placed as for lithotomy, the surgeon makes two semilunar incisions, which comprehend a portion of skin, and unites them at about an inch before and behind the rectum. The skin is dissected up, so as to isolate the rectum on all sides. The advantage thus derived is, that the gut can be forced or drawn down about an inch when the index finger is introduced. When the cancer is superficial, when it is confined to the mucous membrane, or does not extend behind the intestinal tunics, and at the same time does not extend higher than an inch above the anus, it is easy, by bending the index finger in the rectum and drawing downwards, to invert the intestine and expose the whole disease. Then it will be sufficient to divide the inverted portion of the intestine, and to extirpate the diseased part by means of curved scissors.

When the cancer has involved the whole of the tunics of the intestine and the surrounding cellular tissue, when it ascends two or three inches, this method cannot be adopted. It is now necessary, after the preliminary dissection of the inferior extremity of the rectum, to make an incision, parallel to its axis, by means of straight scissors directed on the index finger. This incision should be made on the posterior part, to the limits of the disease. It will allow the intestine to come down, and will expose the whole extent of the disease. This descent is to be maintained by two or three tenacula (airignes.) When a female is operated on, an assistant must render the recto-vaginal septum tense by means of two fingers, introduced into the vagina. In the male, the urethra must be rendered prominent by means of a sound. After these precautions, a dissection must be made of the anterior part of the rectum; it must be made with great slowness and precaution, to avoid perforating the gut. When the anterior part is detached, the rest of the circumference is easily separated, as being more loosely connected; after which the excision above the diseased portion may be made with curved scissors. In these cases the operation is more difficult in the male than female, as it is difficult to separate the intestine from the prostrate, and to render the urethra sufficiently prominent by means of the sound. In both sexes the dissection, however, is difficult and laborious. Vessels are tied as soon as they are divided. Lisfranc does not think the internal pubic need be injured. It should be observed, that when the lower part of the rectum has been divided, it is sometimes drawn up so high that it is difficult to seize it with the fingers and bring it downwards.

After the operation, Lisfranc allows of the oozing of blood so long as the patient is not threatened with syncope; from the formation of coagula, the hemorrhage ceases in one or two hours. The dressings consist of a perforated compress covered with cerate, of lint compresses, and a T. bandage. In females, the urine must be removed by means of a catheter, for fifteen or twenty days, that it may not flow on the wound. Towards the third day, suppuration commences; it has the fetid odour of a stercoraceous abscess: it becomes very copious, and does not diminish until three or four weeks. The cicatrix is formed rapidly, so far as the skin is concerned; afterwards it advances slowly. When the opening begins to contract, it is requisite to introduce a large bundle of lint, and great precautions are requisite that it should penetrate the cavity of the intestine. When the division is high up, the index finger must be employed. A cure is rarely obtained in less than two months, and the tents must be continued for three or four weeks longer. Incontinence of fœces seldom occurs. A kind of annular swelling forms at the extremity of the rectum, which operates as a sphincter; hence the stercoraceous matters, when somewhat hard, having passed this swelling, can remain in the inert canal of the cicatrix, whence the patient can extract them by means of the finger; a slight inconvenience. One patient was for a long time obliged to employ a tampon of lint, which he removed when he went to stool.

Hemorrhage is a rare accident; should it occur, and syncope be threatened, the tampon of lint must be used, if the arteries cannot be secured by ligature, but as the operation of stuffing the rectum is painful and irritating, the lint must be removed in two or three hours. This time will suffice; should it not, the lint must be reapplied.

A series of nervous symptoms very often are manifested immediately after the operation; some of them are troublesome. M. Lisfranc, has employed antispasmodics, narcotics, &c., without any decided effect. He has performed the operation nine times, six upon females, three upon men; of these patients six recovered and three died, viz. two females and one man. Two post mortem examinations were made; an infiltration of pus was found in one case; in the other, phlebitis.—*Ib.*

16. *Ununited Fracture of the Femur successfully treated by the Seton.*—The evidences of the value of this mode of treating ununited fracture, are constantly accumulating. In the *London Medical and Surgical Journal* for December last, the following case is related by John Swift, Esq. A healthy young man, aged twenty-eight, a laborer, was admitted into Richmond Hospital, Dublin, on the 18th January, with a false joint, formed at the junction of the middle and lower thirds of the femur. He stated that about two years and a half since, he had his thigh bone broken by a horse which he rode falling on him. The fracture was simple, but very oblique, and the shortening of the member considerable. In some short time after the accident, he went into one of the provincial hospitals, where the limb was kept in the extended position on a softish bed for two months. When allowed to get up, he found that the first time he attempted to bear his weight on the limb, the fractured portions were not united. He was again confined to bed in the same position, for three months, at the end of which no union had taken place, and he was permitted to walk about on crutches, and discharged, after twelve months confinement in hospital, with a false joint. He had been in Stevens's Hospital subsequently some months, and had glue bandages and splints applied, and was put under the influence of mercury with some benefit. A seton was passed between the fragments, which was followed by considerable inflammation and a copious discharge. At the end of the month it was removed, a complete union having taken place, and in a few days the patient began to bear gently on the limb, and was discharged cured, about six weeks after his admission. He returned again on the 10th of July, with the limb flexible, in the situation of the fracture, and unable to sustain his weight. He stated that being discharged, he employed it very much in digging and walking, being, as he expressed himself, so proud of his recovery, that he thought he could not use the limb often enough. By the aid of rest, full diet, a glue bandage, splints along the limb, and the iodine lotion, the thigh is becoming gradually firmer. He is at present walking about with the assistance of a stick.

We may here remark, that in enumerating in our last No. the cases of ununited fractures successfully treated by the seton, we omitted to notice a case of ununited fracture of the humerus, cured by this measure, related by Dr. Robert Baxter, in the New England Journal of Medicine, vol. VII. p. 150.

It must be truly gratifying to the ingenuous surgeon who devised this mode of treatment, to learn the success which has so frequently attended its employment, and the amount of human suffering, which has thereby been saved.—*Amer. Journ. of Med. Scienees.*

17. *Removal of the Penis by Ligature.*—In our last number we described Professor Graefe's method of amputating the penis by the ligature, and alluded to some cases related by Dr. Michaelis, in the *Journal der Chiruigie and Augenheilkunde*, Band XIII. Stuck 2, in which this operation had been successfully practised. We confess we cannot understand how a ligature can be placed on so sensible a part with so little pain to the patient as is said to have been experienced in these cases. We give an abstract of them as related in the Journal alluded to.

Case 1.—A man aged twenty-nine, affected with a venereal ulcer of the glans, had at first recourse to a mercurial treatment, under which the disease rapidly disappeared, but it reappeared some months afterwards, and resisted every measure. The ulcer affected one-third of the glans, and penetrated as far as the fossa navicularis. The patient was a prey to a mercurial fever, which had reduced him to an extreme degree of emaciation. The mercurial affection was relieved, but the ulcer did not cease to advance. Finally at the end of a year he went to Berlin, and applied to M. Graefe, who removed the diseased part by a ligature. He introduced into the urethra a silver sound, afterwards he strangled the penis immediately behind the corona glandis, be a ligature drawn very tight. The patient supported this operation with the utmost indifference. The same night the part beyond the ligature was insensible and the ligature could be drawn tighter without producing any pain. The second day thediseased part was detached and the sound was withdrawn; the cure was rapid, and the patient left Berlin in about a month. Case II. was that of a man aged thirty-five years, with a carcinoma, which occupied the half of the penis. The operation was performed as in the preceding case, and the patient suffered but little pain. At the end of twenty-four hours the strangulated part was removed. Case III. a man aged sixty-two, with carcinoma of the penis originating in the prepuce. No accident resulted from the operation, and the wound cicatrized so promptly that the patient left the hospital on the 10th day. The fourth case was also one of carcinoma. It occupied nearly the whole of the penis. The patient was perfectly cured by the ligature in about fifteen days. —*American Journ. of Med. Sciences.*

18. *Poisoning by Mouldy Bread.*—Dr. Westerhoff was called, in 1826, to two children of a poor laborer, who had been simultaneously attacked with the following symptoms. The eldest, aged ten years, had his face red and swollen, his countenance was animated and bewildered, tongue dry, pulse feeble and quickened, head-ache, giddiness, unextinguishable thirst, violent colic, desire to sleep, and alternate unsuccessful efforts to vomit, subsequently sudden vomiting and very abundant alvine evacuations, after which very great faintness, indifference to every thing, and sleep for moments at a time. The younger, aged eight years, was rather more violently attacked than his brother. Dr. W. having understood that they had eaten, the preceding day, only a piece of old, mouldy, rye bread, prescribed a demulcent treatment, and they soon recovered.

Some time afterwards, several boatmen having eaten some mouldy rye bread, were attacked with similar symptoms, but they were quickly relieved by vomiting, which came on spontaneously. Dr. W. asks whether this kind of poisoning arises from an alteration in the quality of the bread, or from the vegetation which has been called mouldiness.—*Archives Generales.*

19. *Case of Spasmodic Asthma relieved by Inhalation of Ether, Conium, and Ipecacuanha.*—A married lady, aged thirty-six, had been subject to attacks of spasmodic asthma for some years past, from which she obtained relief by the use of antispasmodic and expectorant medicines; but her stomach was often disordered by their influence, and she had recourse to them with reluctance. I was desirous of trying the comparative power of inhalation, and prescribed, for this purpose, a mixture consisting of æther, conium, and ipecacuanha. I subjoin a statement of its effects in the words of the intelligent patient.

"I inhaled the medicated vapour during fifteen minutes before going to rest. The first sensations it occasioned me, were slight fatigue in breathing, and an aching pain in the breast, which, however, subsided by degrees; and when expectoration took place, which occurred copiously within half an hour after the inhalation, I felt completely relieved. Afterwards in the course of the night, whenever I awoke, (instead of feeling the oppression and the difficulty of breathing which often distresses me,) expectoration, without effort took place, and breathing easily and freely, I then slept again immediately. Usually, whenever I awake with the sensation of tightness across the chest, I do not sleep for an hour or two afterwards.

"During two days after the inhalation, slight expectoration continued; and ever since, (now ten days,) my breathing, both night and day, has been perfectly free."—*Scudamore on consumption.*

20. *Death from Inhaling Nitrous Ether.*—The only case of this kind we recollect to have seen recorded is in the *Midland Medical and Surgical Reporter*, for August last. The subject of this was a case female servant; she was found dead in her bed, lying on her right side, with her arms folded across the breast as in a profound sleep, and the features not at all disturbed. On examination, "the coats of the stomach was found a little inflamed, with a small quantity of fluid in it, not exceeding one ounce—there was no gritty substance in this organ. The intestines leading from the stomach appeared turgid." The lungs were in such a high state of congestion as to prevent the passage of air through the cells. A large jar containing upwards of three gallons of spirits of nitrous ether, was found in the patient's room, broken, and the the contents spilled, and the apartment being small, the atmosphere was highly impregnated by the spirit, which was no doubt the cause of death.

It is much to be regretted that the examination of the body was confined to ascertaining the state of the lungs and stomach. The condition of the brain, the heart, and of the whole alimentary canal; the state of the blood, and the appearances of the surface of the body should have been described.—*Glasgow Med. Journ.*

*Test for distinguishing different kinds of Rhubarb.*—According to M. Geiger, the ioduretted hydriodic acid produces different colours in different kinds of rhubarb, which enables us to distinguish them. Thus it gives to Muscovy rhubarb a green tinge—to China rhubarb a brown tinge—to English rhubarb a deep red color, and to that of France a blue one.—*Journal de Chimic Medicale, Sept.* 1830.

21. *New operation for Ectropion.*—Dr. J. F. Dieffenbach, of Berlin, has described, in a late number of *Rust's Magazin*, the following operation for the permanent cure of everted eyelid. A semilunar incision is first made through the skin, a few lines within the edge of the orbit. This incision is to be made directly in the centre of the lid, and should occupy about two-thirds of its extent. The lip of the wound next the tarsus, is to be dissected up, so as to loosen a considerable portion of the everted tarsus, when the whole thickness of the lid is to be cut through to the extent of the external wound. A small pair of forceps being now introduced into the wound, that portion of the conjunctiva to which the tarsus is attached, is to be drawn out at the external orifice; and the edges of the wound, together with the retracted conjunctiva, are to be held together by means of from three to five small needles, over which a thread is to be passed, as in the hair-lip suture.—*Ed. Med. and Surg. Journ.*

22. *Opacities of the Cornea.*—Dr. Shortt says that he is inclined to believe that, in one or two instances, strychine applied for the cure of amaurosis, was beneficial in removing opacities of the cornea, probably by its highly stimulating property occasioning rapid absorption. —*Ib.*

23. *Cure of Subcutaneous Nævus by the Seton.*—Mr. Fawdington, of Manchester, has published in the North of England Medical and Surgical Journal, for August last, three cases of nævus cured by seton. He advises the remedy in cases where the size of the tumour precludes the use of the knife, caustic, or ligature, and states that it will be used with more success than tying the artery which supplies the tumour, and that it is followed by scarcely any disfigurement. The skein of thread should be large enough to fill up the apertures made by the needle, and thus to arrest hemorrhage, and, by using this precaution, a sufficient degree of irritation will be produced to excite inflammation and suppuration throughout the diseased mass. The first case was that of a fine male infant, about three years and a half old, who had a nævus between the angle of the jaw and mastoid process, extending upwards to the zygomatic arch. The whole formed an oval tumour, which measured five inches and a quarter in its long axis, and four inches transversely. It had no pulsation, was purplish, soft, and compressible, and had large veins on its surface. A skein of common sewing thread was passed through it with a saddler's needle, and no dressings were applied. On the third day the tumour was inflamed, and on the sixth, in a state of suppuration; on the tenth, resembled the site of an abscess or common bile, and at one part but a portion of the tumour remained, through which a seton was passed with similar results. In four months there was not a vestige of the original disease.

The second case was that of an infant ten months old, who had a nævus on the forehead. A seton partially removed it; a solution of sulphate of copper was applied, which produced inflammation and suppuration, but a second seton was required to complete the cure.—*Lond. Med. and Surg. Journ., Sept.*, 1830.

24. *On Lithoplatomy.*—By M. S. Buchanan, M. D.—Case I. Mrs. Grant, aged 50, of full habit of body and lax fibre. Has been subject, for many years, to calculous complaints, but, till lately, would never submit to examination. On the 29th of July last, was admitted, under my care, into the Royal Infirmary, and calculi of various sizes with ease detected lying at the neck of the bladder. In addition to the usual symptoms attendant on this painful disease, she had the most complete incontinence of urine I ever saw, the bladder having become much contracted, its coats thickened and constantly discharging white ropy mucous from its internal surface. Extensive excoriation of nymphæ, labia, nates, and thighs, was the consequence, and considerable paralysis of inferior extremities had of late rendered her a most pitiable object.

Immediately on admission, I ordered her bowels to be well opened, anodyne injections to be administered, and the warm bath to be used; and, on the 3d day of August, with consent of consultation, I dilated the urethra with Weiss' instrument, and extracted, with the aid of a small pair of forceps, three stones, two of them about the size of filberts, and the third about the size of a walnut. She experienced little pain during the process of dilatation, which occupied about seven minutes. The incontinence of urine, and consequent excoriation of nates, &c., in a short time disappeared; and when she left the house, about three weeks after the operation, she could walk from one end of the ward to the other without assistance, her general health having at the same time greatly improved.

Case II. Robert Brock, a gentleman's servant, was admitted, under my care, into the Infirmary, on the 11th of August last. At that time a stone could be felt in the urethra, a little anterior to the bulb. It had advanced thus far about a fortnight previous to admission, and, from its position, now caused excruciating pain. He stated, that from his infancy he had labored under symptoms unequivocally indicating stone in the bladder, but till lately would not consent to be sounded. This operation was repeated with great care by several surgeons in this city, some months previous to admission, without any stone having been detected; and various means were also adopted, the fortnight prior to the 11th instant, to get the stone dislodged from its situation in the urethra, by the introduction of catheters and bougies of the largest size, to the spot where it seemed sacculated; and also by the trial of various kinds of urethral forceps, assisted by the warm bath, &c., but all to no purpose.

The gentlemen who met in consultation on his admission, were so convinced that no further trials of the above kind would succeed, that he was immediately placed on the operating-table, secured as for lithotomy, a grooved staff introduced as far as the stone, and, with one stroke of the knife, the urethra was incised to the proper extent, and the stone extracted. The operation was the affair of a moment, and was so free of pain, that the patient seemed astonished at having handed to him the cause of all his misery.

The stone measured two inches and eight lines in its largest circumference, and one inch and ten lines in its smallest; it was convex on its one side, and flat on the opposite, with a small nodule in the centre of this last, indicating that it was the smaller segment merely of a larger stone, which must still be in the bladder. This was made more evident, not only from the very sharp edge of the flat surface above described, (which had very much the appearance of having been recently broken,) but also from the concentric layers of lithic acid, which were observed when sawn through its largest diameter by the lathe. On the 14th, the wound in the perineum was completely healed, and no symptoms of calculus remained. He has been frequently sounded since, without any stone having been discovered, and, in consequence of feeling in every respect well, he was dismissed cured.

*Query* 1st. Is there any instance on record in which a stone of such dimensions was passed thus far into the urethra from the bladder?

2d. In what manner shall we account for the section or fracture of this stone in the bladder, no instrument having at any time detected it till its exit from this viscus?

3d. Why is the alleged remaining half unable to be discovered by the most careful sounding, as the first was, previously to its escape from the urethra?

But if, notwithstanding all that has been advanced, it is denied that the male urethra is capable of dilatation in the manner suggested, another method remains to be discussed, of more importance by far than any to which I have hitherto adverted, and to which I am inclined more particularly to apply the term lithoplatomy.

The operation I allude to is that by which the male urethra is converted into a female one, and thus the difficulty and danger of both lithotomy and lithotrity is, at one step, superseded by lithoplatomy.

For the purpose of effecting the first part of the operation, the patient must be secured as for lithotomy; a grooved staff is introduced into the bladder; the membranous part of the urethra near the prostate incised, to the necessary extent, to admit the lithoplatome; and then, having withdrawn the staff, the operation of dilatation of the remaining part of the urethra, prostate, and neck of the bladder, will be, with nearly the same ease, safety, and effect, accomplished by means of my lithoplatome, as in the female.

That there are many objections to the above method of operating for stone, I must at once admit; and, until I can give it a fair trial on the living subject, I shall not contrast it either with lithotomy or lithotrity,—judging, however, from what I have already experienced in the female urethra, and, on the dead subject, in the male, I am not the least afraid of the result. I shall only add further, that, if the operation of lithoplatomy does come up to my expectations, and that, of many judicious and talented surgeons here, to whom I have mentioned it, how much will suffering humanity be relieved, and the labors of the surgeon facilitated.

But lest it should be said that the operation of lithoplatomy which I have above described, and so strongly recommended, is nothing more than the apparatus major, the Marian method, that of Le Cat, Le Drân, or Pajola, let any one take a glance at these methods of operating, and the difference must at once strike him. * * * It is the principle, I once more repeat, of *slow* and *continued dilatation*, by the *instrument* which I have recommended, applied to the *whole* or a *small part* of the *male urethra*, for the extraction of calculi, which alone constitutes my improvement of this part of surgery.

In stricture of the urethra, could a very slender lithoplatome, introduced through the constriction, not be made to overcome this more safely and more speedily than either the simple or armed bougie, or conical sound? If the instrument cannot be passed through the spasmodic or organic contraction of this canal, could it not, by its introduction as far as the stricture, and kept steadily in its place, be made, by gentle dilatation, to overcome this disease, or at least allow of armed bougies, or sounds, being more safely made to act in accomplishing their object?—*Bost. Med. and Surg. Journ.*

25. *Observations on some Cases of supposed Rheumatic Affection of the Lower Extremities in Females*, by Madame Boivin.—It will be some time before we see such minute pathological investigations from the pen of a female writer in this country, as Madame B. occasionally sends forth into the medical world! But we shall let the lady speak for herself.

Case 1. A female, fifty-five years of age, the mother of seven children, had always enjoyed good health till the age of fifty-two, when she was seized with pains in the iliac regions, and in the lower extremities The catamenia had been suppressed sometime previously. Leeches, frictions, anodynes, warm baths, and various other means were used, without any relief. The menses returned; but the pains persisted. They were considered Rheumatic. A discharge of blood now took place from the uterus, and continued more or less, for many months, without exciting any suspicion in the mind of the medical attendant, till Madame Boivin and M. Dubois were consulted, when they discovered an enormous cancer of the uterus, together with an ovarian disease causing adhesions about the pelvis, which no doubt gave origin to the pains considered rheumatic. She was sent from the *Maison de Sante* into the country to die.

Case 2. This case shows the danger of a too speedy return to occupations after an accouchment.

A female did not menstruate till the age of twenty; but, after that period, had the catamenia regularly continued till her 25th year, when she became pregnant, and went into the hospice of the Ecole de Medicine for accouchement. Her delivery was rapid, the labor only continuing one hour. On the *third* day, the female returned to her master's house, and took to her usual avocations of cooking. But she was soon seized with violent fever, swellings of the mammæ, pains in the abdomen, in the left iliac region, to which leeches and fomentations were applied. She was sent into the hospital Necker, where she was properly treated, and comparatively cured; but still there remained a tumor in the left iliac region, with pains in the hip and thigh, which were considered rheumatic by her subsequent medical attendants. She ultimately came under Madame Boivin's observation in a *Maison de Sante*, and on accurate examination, the os uteri was found to be thrown out of its proper situation; to be hard and enlarged; while a tumor was felt occupying the iliac region, which was considered as a diseased ovarium. By proper treatment the symptoms were much mitigated; but she was discharged uncured.

Several other cases are related by Madame Boivin, to show how frequently medical men confound the pains resulting from organic disease in the pelvis, with rheumatic pains in the lower extremities. These we need not detail. The above are sufficient to put practitioners on their guard.—*Medico Chirurg. Review.*

26. *Camphor.*—Libri has mentioned a curious circumstance regarding odoriferous bodies, such as camphor; that if they be exposed to a current of electricity for a considerable time, their smell diminishes, and at last disappears entirely. After a lapse of time, camphor again recovers the power of emitting odors.—*Edenb. Jour. of Science.*

27.—*Economic Lighting.*—At the Tulloch Bleachfield, a young man named A. Reed, has constructed an apparatus, by which he is enabled to procure from the wood, which they are in the practice of burning, in order to obtain acetic acid, gas sufficient to light the whole premises. By this ingenious device, a most important saving is effected—since no more wood is necessary for both the gas and the acid, than was formerly used for the acid alone.—*Bost. Med. and Surg. Jour.*

